# Predictive role of UCA1-containing exosomes in cetuximab-resistant colorectal cancer

**DOI:** 10.1186/s12935-018-0660-6

**Published:** 2018-10-22

**Authors:** Ying-nan Yang, Rui Zhang, Jing-wen Du, Heng-heng Yuan, Yan-jing Li, Xiao-li Wei, Xiao-xue Du, Shu-lin Jiang, Yu Han

**Affiliations:** 1 Department of Thoracic Surgery, Harbin Medical University Cancer Hospital, Harbin, Heilongjiang China; 2 Department of Colorectal Surgery, Cancer Hospital of China Medical University, Liaoning Cancer Hospital & Institute, Shenyang, Liaoning China; 3 Department of Gastrointestinal Oncology, Harbin Medical University Cancer Hospital, 150 # Haping Road, Harbin, 150081 Heilongjiang China; 4 Department of Cardiovascular Surgery, The Second Affiliated Hospital of Harbin Medical University, 246 # Xuefu Road, Harbin, 150001 Heilongjiang China

**Keywords:** UCA1, Exosomes, Cetuximab, Metastatic colorectal cancer, Biomarker

## Abstract

**Background:**

Primary or acquired resistance to cetuximab often occurs during targeted therapy in metastatic colorectal cancer (mCRC) patients. In many cancers, the key role of the long noncoding RNA (lncRNA) urothelial carcinoma-associated 1 (UCA1) in anticancer drug resistance has been confirmed. Emerging evidence has shown that specific exosomal lncRNAs may serve as meaningful biomarkers. In this study, we hypothesize that exosomal UCA1 might predict the response to cetuximab in CRC patients.

**Methods:**

First, acquired cetuximab-resistant cell lines were generated, and UCA1 expressions in these cells and their exosomes were compared. We also systematically evaluate the stability of exosomal UCA1. Thereafter, the predictive value of exosomal UCA1 in CRC patients treated with cetuximab was evaluated. Finally, through cell apoptosis assays and immunofluorescence staining, we analyzed the role of UCA1-containing exosomes in conferring cetuximab resistance.

**Results:**

UCA1 expression was markedly higher in cetuximab-resistant cancer cells and their exosomes. Exosomal UCA1 was shown to be detectable and stable in serum from CRC patients. In addition, circulating UCA1-containing exosomes could predict the clinical outcome of cetuximab therapy in CRC patients, and UCA1 expression was considerably higher in the progressive disease/stable disease patients than in the partial response/complete response patients. Furthermore, exosomes derived from cetuximab-resistant cells could alter UCA1 expression and transmit cetuximab resistance to sensitive cells.

**Conclusions:**

We discovered a novel role of UCA1-containing exosomes, showed their capability to transmit drug resistance and investigated their potential clinical use in predicting cetuximab resistance.

## Background

Colorectal cancer (CRC) is a leading cause of cancer-related death worldwide [1]. Screening, surgery, and medical therapies are usually effective for the management of early-stage CRC, however, these treatment options are far less effective for advanced-stage disease. Cetuximab is an epidermal growth factor receptor (EGFR) monoclonal antibody (mAb) that binds the EGFR extracellular domain and enhances receptor internalization and degradation. This mAb targeting EGFR is a common targeted agent used to treat patients with metastatic CRC (mCRC) with wild-type KRAS status [2]. However, patients frequently develop drug resistance. A number of genetic biomarkers, including RAS (KRAS exons 3 and 4 or NRAS exons 2, 3, and 4) mutations [3, 4], BRAF (V600E) [5], PIK3CA (exon 20) [6] and HER2 and MET amplification [7, 8], are robustly correlated with cetuximab resistance. Despite these genetic biomarkers, additional mechanisms of resistance to EGFR mAb are thought to be present in mCRC.

Currently, with advancements in global transcriptome profiling technique, the functional roles of long noncoding RNAs (lncRNAs) have received considerable attention in human cancer [9]. LncRNAs are mRNA-like transcripts ranging in length from 200 nucleotides (nt) to over 100 kilobases (kb) lacking significant protein-coding ability [10]. Emerging evidence has demonstrated that lncRNAs play significant roles in biological processing, including cell cycle regulation, apoptosis and tumor invasion [11]. Additionally, other lncRNAs have been shown to play functional roles in resistance to targeted therapy [12]. Some lncRNAs could be potentially used as early diagnostic, prognostic and drug response biomarkers in malignant tumors [12–14].

Urothelial carcinoma-associated 1 (UCA1) is a lncRNA with three exons, and several recent studies have demonstrated oncogenic functions of UCA1 in various types of cancer, such as breast, bladder, colorectal, and gastric cancer [15]. In our previous study, we elucidated that UCA1 expressions are significantly increased in CRC tissues and cells, and this high UCA1 expression level is significantly correlated with larger tumor size, greater tumor depth and less differentiated histology. Additionally, patients with high UCA1 level have a significantly poorer prognosis than those with low UCA1 level. Moreover, UCA1 influences the proliferation, cell cycle and apoptosis of CRC cells. These data indicate an important role for UCA1 in the molecular etiology of CRC and implicate a potential application for UCA1 in CRC diagnosis, prognosis of tumor progression, and therapy [16]. In addition to its oncogenic function, UCA1 regulates drug resistance in some kinds of malignant tumors [17]. However, the value of UCA1 levels in body fluid samples regarding the response of CRC patients to cetuximab remains to be confirmed.

Exosomes are lipid vesicles with a diameter of 30–100 nm that are secreted by the fusion of multivesicular bodies with the plasma membrane or by budding from the membrane [18]. Nucleic acids, proteins and lipids are loaded in exosomes, thereby allowing the transfer of genetic material and enabling the exchange of information between cells. Emerging evidence has demonstrated that some specific exosomal lncRNAs expression is correlated with cancer patients’ clinicopathological characteristics and therefore may act as a potential biomarker [18–20]. Based on these observations, we hypothesize that exosomal UCA1 might predict the response of CRC patients to cetuximab.

The existence and stability of exosomal UCA1 in serum were systematically investigated in this study. Additionally, we identified a novel role of UCA1-containing exosomes in predicting the response of CRC patients to cetuximab. Moreover, for the first time, we suggest that UCA1 may be transmitted via exosomes, thus affecting drug metabolism.

## Methods

### Patients and sample processing

Informed consent was obtained from each patient, and the protocols were approved by the Ethics Committee of Harbin Medical University Cancer Hospital. Serum samples were collected from Harbin Medical University Cancer Hospital to investigate the feasibility of detecting exosomal UCA1. Changes in serum exosomal UCA1 levels were validated in an independent cohort of 53 CRC patients treated with cetuximab at Harbin Medical University Cancer Hospital from 2015 to 2016. Blood samples from all CRC patients were collected by vena puncture. To extract exosome from human blood, a two-step centrifugation protocol—1600*g* for 10 min and 16,000*g* for 10 min at 4 °C was used to isolate serum, and then serum was stored at − 80 °C until exosome extraction. Blood samples with evidence of hemolysis were excluded. According to the RECIST criteria for a pathological response, these patients were divided into two groups: 30 patients responded to cetuximab therapy [complete response (CR) or partial response (PR)], and 23 patients did not respond [stable disease (SD) or progressive disease (PD)].

### Cell lines and culture

The human Caco2 cell line was purchased from the Cell Bank of the Chinese Academy of Sciences (Shanghai, China). We established cetuximab-resistant cell lines by chronically exposing cetuximab-sensitive Caco2 (Caco2-CS) cells to increasing cetuximab doses in medium over a period of 6 months. The final concentration of cetuximab for the cetuximab-resistant subclone Caco2-CR was 300 μg/ml. Caco2-CS and Caco2-CR cells were cultured in Dulbecco’s modified Eagle’s medium (DMEM, Gibco, Invitrogen, USA) containing 10% fetal bovine serum (FBS, Gibco, Invitrogen, USA) and 1% penicillin–streptomycin (Invitrogen, USA) at 37 °C in a humidified atmosphere of 95% air/5% CO_2_.

### Cell proliferation assay and colony formation assay

For the cell proliferation assay, cell viability was determined by Cell Counting Kit 8 (CCK8, Dojindo, Japan) according to the manufacturer’s instructions. For the colony formation assay, about 1000 cells were placed in each well of a 6-well plate in 2 ml media containing cetuximab (300 μg/ml for Caco2-CR). The media were changed every 3 day. After 12–15 days, the colonies were fixed in 80% methanol and stained with 0.1% crystal violet for 20 min. The number of colonies was counted using an inverted microscope.

### Isolation of exosomes

Medium and serum were filtered through a 0.45 μm polyvinylidene fluoride filter (Millipore, Billerica, MA, USA); ExoQuick solution (System Biosciences, Mountain View, CA, USA) was added to the serum, which was then incubated for 0.5 h at room temperature, and ExoQuick-TC solution was added to the medium, which was then incubated at 4 °C for 12 h. The mixture was centrifuged at 1500*g* for 30 min and supernatant was removed by aspiration. Pelleted fractions were resuspended in 25 μl phosphate-buffered saline (PBS).

### Transmission electron microscopy (TEM)

A sample of exosomes was diluted to a final concentration of 0.5 mg/ml in PBS, spotted onto a glow-discharged copper grid on filter paper and dried for 10 min. Exosomes were stained with 1% aqueous phosphotungstic acid (PTA) for 5 min and dried for 20 min and then examined at 100 keV with TEM (JEM-1-11 microscope, Japan).

### RNA extraction

Total RNA was extracted from cells using TRIzol^®^ Reagent (Invitrogen, CA, USA). Exosomal RNA was extracted using the Total Exosome RNA and Protein Isolation Kit (Invitrogen, USA). The concentration and quality of RNA were measured by UV absorbance at 260 and 280 nm (260/280 nm) using a Nanodrop 2000 spectrophotometer (Thermo Scientific, USA).

### Quantitative real-time reverse transcription-polymerase chain reaction (qRT-PCR)

Total RNA was extracted from cells and exosomes as described above. RNA templates were treated with DNase I to avoid genomic DNA contamination. The first strand of cDNA was synthesized using the SuperScript First-Strand Synthesis System (Invitrogen, CA). PCR amplification was performed using an Applied Biosystems 7500 Detection System (Applied Biosystems, CA) and primers for UCA1 (forward: 5′-ACGCTAACTGGCACCTTGTT-3′, reverse: 5′-TGGGGATTACTGGGGTAGGG-3′) and β-actin (forward, 5′-CACCTTCTACAATGAGCTGCGTGTG-3′; reverse, 5′-ATAGCACAGCCTGGATAGCAACGTAC-3′). Real-time PCR was performed on triplicate samples according to the instructions of the SYBR^®^ Premix Ex Taq™ Kit (Takara, Japan). The expression level of UCA1 was normalized to that of β-actin using the comparative 2^−ΔΔCt^ method.

### Western blot analysis

Proteins were extracted from cells using RIPA lysis buffer (Biouniquer Technology, China). Exosomal proteins were extracted using the Total Exosome RNA and Protein Isolation Kit (Invitrogen, USA). Protein content was measured using a Nanodrop 2000 spectrophotometer (Thermo Scientific, USA). Equal amounts of protein from each sample were separated by electrophoresis in sodium dodecyl sulfate (SDS)-polyacrylamide gels before being transferred onto polyvinylidene difluoride (PVDF) membranes, blocked for 2 h with 5% skim milk, incubated overnight at 4 °C with primary antibodies against TSG101, Alix and CD81 (Abcam, Cambridge, UK) and then incubated for 1 h with the secondary antibody (Kangwei Ltd., China). The signals were visualized with an ECL detection system.

### Immunofluorescence assay

For exosomal labeling, Dil (Beyotime Biotechnology, China) was added to the exosome suspension at 1 μM for 20 min, and the exosome suspension was then washed through Exosome Spin Columns (MW3000) (Invitrogen, USA) to remove excess dye. Dil-labeled exosomes were incubated with CRC cells and visualized by fluorescence microscopy or EPICS XL flow cytometry (Beckman Coulter) after 24 h.

### RNA interference

Caco2-CR cells were transfected with UCA1-small interfering RNA (siRNA) (RNAi: forward primer: 5′-GCACCUUGUUAGCUACAUAAA-3′, reverse primer: 5′-UAUGUAGCUAACAAGGUGCCA-3′) using Lipofectamine™ 2000. After 48 h of transfection, RNAi efficiency was assessed by qRT-PCR. Another siRNA was designed as a negative control (Ctrl siRNA): forward primer: 5′-UUCUCCGAACGUGUCACGUdTdT-3′, reverse primer: 5′-ACGUGACACGUUCGGAGAAdTdT-3′.

### Electroporation of UCA1 into exosomes

UCA1 was electroporated into exosomes using a GenePulser Xcell™ electroporation system (Bio-Rad, USA) as previously described [21]. Briefly, 2 μg exosomes and 400 nmol RNA were mixed in 400 μl electroporation buffer and electroporated at 350 V and 150 μF in a 4-mm cuvette. The mixture was incubated at 37 °C for 30 min to ensure that the exosome membrane fully recovered, and then, the mixture was treated with RNase to remove unincorporated RNA. Labeled RNA in exosomes was quantified by detecting fluorescence using a Fluorescence Spectrophotometer F-4600 (HITACHI, Japan).

### Apoptosis assay

Twenty-four hours after 1 × 10^5^ Caco2-CS cells were seeded in six-well plates, different amounts of CR-exo (1, 5, or 10 μg) were added to the supernatants. The samples were divided into the following four groups: CS, CS + 1 μg CR-exo, CS + 5 μg CR-exo, and CS + 10 μg CR-exo. After culturing for 24 h, the cells were treated with cetuximab (300 μg/ml) for 48 h. Then, all the cells were harvested by centrifugation, washed with PBS, stained using Annexin V fluorescein isothiocyanate (APC) and propidium iodide (PI) and analyzed using a flow cytometer (BD Biosciences, San Diego, CA).

### Statistical analyses

All statistical analyses in this study were performed with SPSS 16.0 software. All experiments were carried out three times independently. Data are presented as the mean ± standard deviation. p values < 0.05 by Student’s t-test or one-way ANOVA indicated statistical significance. All statistical tests were two-sided.

## Results

### Upregulation of UCA1 levels in cetuximab-resistant cells and their exosomes

**Fig. 1:**
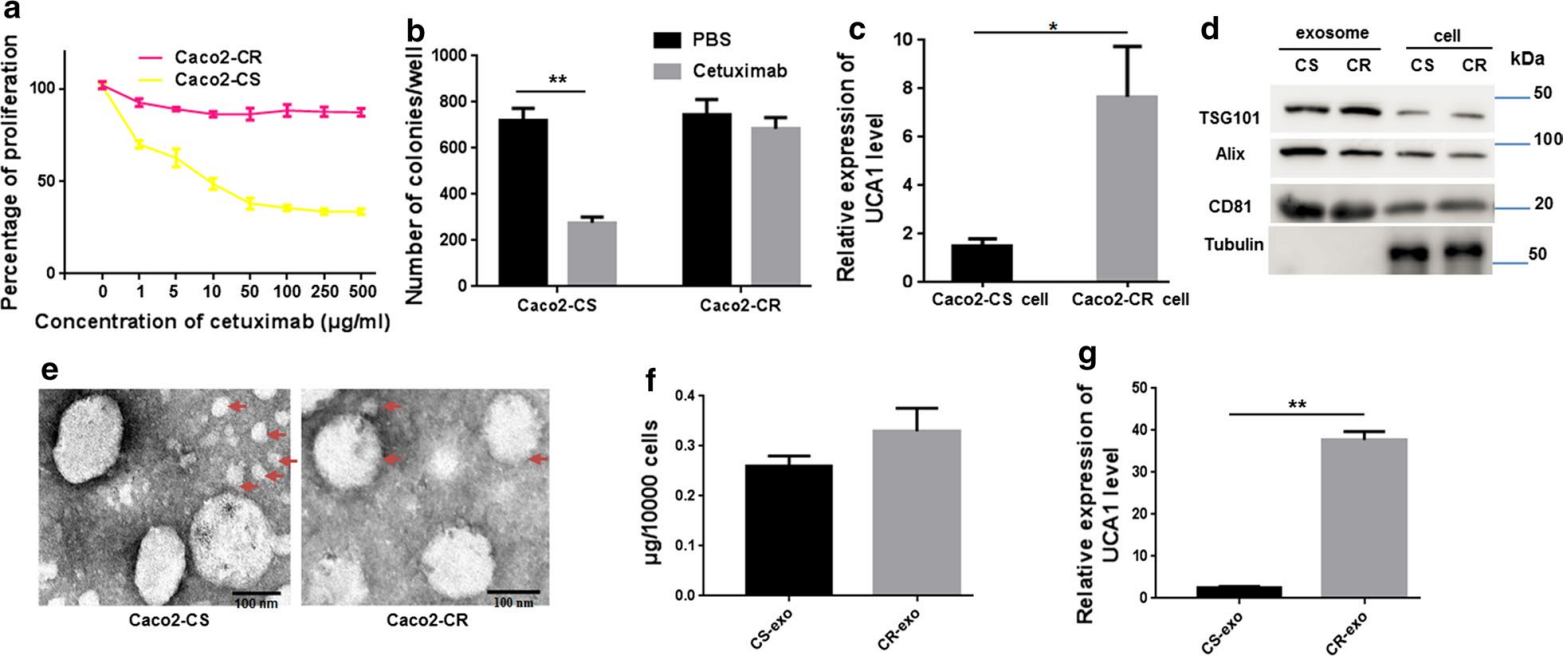
Levels of UCA1 in cetuximab-resistant and cetuximab-sensitive cells and their exosomes. **a** We treated Caco2-CS and Caco2-CR cells with increased concentrations of cetuximab and evaluated the cell proliferation by CCK8 assay. **b** Colony formation assays were performed to determine the effect of PBS or cetuximab (300 μg/ml) on Caco2-CS and Caco2-CR cells colony formation. **p < 0.01. **c** UCA1 levels were significantly higher in Caco2-CR cells than in Caco2-CS cells; *p < 0.05. Data are shown as the mean ± SD. **d** Western blot analyses were used to detect exosome markers (CD81 and TSG101) in Caco2-CS (CS) cells and Caco2-CR (CR) cells. **e** Exosomes were observed by TEM. Exosomes are highlighted using red arrows. Scale bar, 100 nm. **f** Quantification of exosomes expelled from equal numbers of Caco2-CS/CR cells. **g** UCA1 levels were higher in CR-exo than in CS-exo; **p < 0.01

We generated cetuximab-resistant Caco2-CR cells from Caco2-CS CRC cells following long-term exposure to increasing doses of cetuximab. Caco2-CR cell lines could grow in culture medium containing 300 μg/ml cetuximab and displayed a robust cetuximab-resistant phenotype as compared to parental controls (Fig. 1a). Additionally, the clonogenicity of Caco2-CR cells was significantly greater than that of Caco2-CS cells in response to cetuximab treatment (Fig. 1b).

Total RNA was extracted from Caco2-CS and Caco2-CR cells, and UCA1 expression levels were assessed using qRT-PCR. UCA1 expression was markedly higher in Caco2-CR cells than in parental Caco2-CS cells (Fig. 1c). We then isolated exosomes from the conditioned medium of Caco2-CS and Caco2-CR cells. Western blot analysis demonstrated the expression of TSG101, Alix, and CD81, which are all exosome markers and are associated with exosome formation, in both exosomes and cells (Fig. 1d). In addition, TEM revealed that the predominant vesicles were of typical exosome size (30–150 nm in diameter), had a characteristic round or saucer shape, and were delineated by a lipid bilayer (Fig. 1e). Further quantitative analysis suggested that the number of exosomes expelled from both cell variants did not differ significantly (p > 0.05; Fig. 1f). Moreover, we detected UCA1 levels in exosomes derived from Caco2-CR cells (CR-exo) and Caco2-CS cells (CS-exo) and found that UCA1 levels were significantly upregulated in CR-exo compared with CS-exo (Fig. 1g). Interestingly, the increase in UCA1 levels between CR-exo and CS-exo (~ 20-fold) was much greater than that between Caco2-CR and Caco2-CS cells (~ 7-fold), indicating that UCA1 was concentrated in exosomes derived from Caco2-CR cells; moreover, UCA1 expressions may be related to cetuximab resistance in CRC cells.

### Characterization of serum exosomal UCA1

**Fig. 2:**
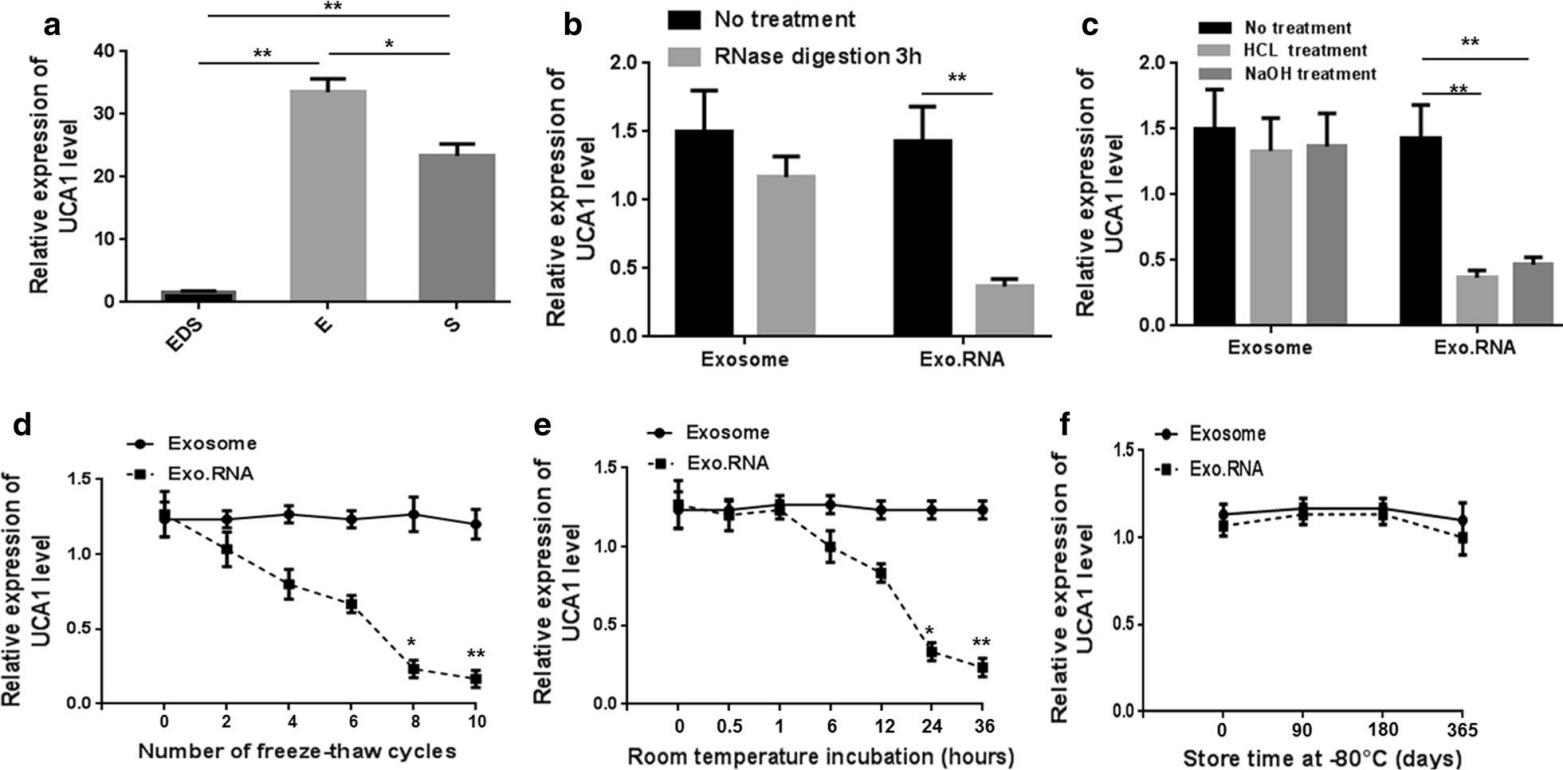
General characterization of exosomal UCA1. **a** Exosomal UCA1 expressions detected from exosome-depleted supernatant (EDS), serum exosomes (E) and whole serum (S). Comparison of UCA1 levels between the exosome group and the isolated RNA (Exo. RNA) group after 3 h of treatment in RNase A (**b**), low pH (pH = 1) solution or high pH (pH = 13) solution (**c**), multiple freeze–thaw cycles (**d**), or incubation at room temperature (**e**) or − 80 °C (**f**); *p < 0.05, **p < 0.01

Figure 2a shows the relative expression of lncRNA UCA1 acquired using the same amount of total RNA. qRT-PCR analysis indicated that the exosomal UCA1 could be reliably detected but outside exosomal UCA1 was only minimally detected. To explore the potential benefit of detecting exosomal UCA1, we skipped the exosome extraction step and extracted RNA from whole serum directly. The data presented in Fig. 2a suggested that UCA1 levels were higher in exosome samples than in whole serum samples (p < 0.05).

To ascertain whether the exosomal UCA1 can be protected by exosome membrane, exosome samples and nucleic acids isolated from exosome were subjected to harsh conditions. Samples were incubated with RNase A, a strong acid, or a strong base for 3 h at room temperature, and the results indicated that RNase A (Fig. 2b) and acid or base (Fig. 2c) treatments had minimal effects on the expression of exosomal UCA1 in the exosome group but caused complete degradation of UCA1 in the isolated nucleic acid group. In the freeze–thaw cycle test, exosomal UCA1 levels showed no significant change but decreased markedly in the isolated nucleic acid group after eight freeze–thaw cycles (Fig. 2d). The levels of exosomal UCA1 in the exosome group were not significantly changed from 0 to 36 h at room temperature, but were markedly decreased in the isolated nucleic acid group after 24 h (Fig. 2e). Moreover, as shown in Fig. 2f, exosomal UCA1 remained stable in both groups at − 80 °C. Collectively, these data suggest that exosomal UCA1 is detectable and stable in exosomes, providing a basis for its use as a predictive biomarker of the response to cetuximab.

### Circulating UCA1-containing exosomes predict the clinical outcome of cetuximab therapy in CRC patients

In order to investigate whether UCA1-containing exosomes could be a useful tumor-derived material for predicting the clinical outcome of cetuximab therapy in CRC patients, we used qRT-PCR to evaluate the features of UCA1-containing exosomes in the peripheral blood from 53 patients who were treated with cetuximab in combination with chemotherapy either as first-line (n = 39, 73.6%) or third-line therapy (n = 14, 26.4%). The main characteristics of all enrolled patients are summarized in Table 1. All subjects were confirmed to have wild-type KRAS (exon 2) before cetuximab administration. Patient’ ages ranged from 31 to 82 years. Tumors were staged according to the American Joint Committee on Cancer (AJCC) 2010 guidelines. Based on patient response to cetuximab, the cohort was separated into two groups: the responder group included 30 (56.6%) patients with either a CR (n = 3, 5.7%) or PR (n = 27, 50.9%), and the nonresponder group included 23 (43.4%) patients with SD (n = 17, 32.1%) or PD (n = 6, 11.3%). Similar response rates were found among the first-line-treated patients (59.0%, 23 of 39) and the third-line-treated patients (50.0%, 7 of 14).

**Table 1:** Characteristics of patients with metastatic colorectal cancer

	All patients n (%)	Responders n (%)	Non-responders n (%)	*p*-value*
Total number	53 (100)	30 (56.6)	23 (43.4)	
Sex				NS
Male	31 (58.5)	18 (60.0)	13 (56.5)	
Female	22 (41.5)	12 (40.0)	10 (43.5)	
Age				NS
≤ 70	38 (71.7)	25 (83.3)	13 (56.5)	
> 70	15 (28.3)	5 (16.7)	10 (43.5)	
Median (range)	56 (31–82)	51.5 (31–82)	59 (37–77)	
Histologic grade				NS
Low grade^a^	45 (84.9)	27 (90.0)	18 (78.3)	
High grade^b^	8 (15.1)	3 (10.0)	5 (21.7)	
Metastatic pattern				NS
Metachronous	14 (26.4)	7 (23.3)	7 (30.4)	
Synchronous	39 (73.6)	23 (76.7)	16 (69.6)	
Primary tumor site				NS
Colon	32 (60.4)	18 (60.0)	14 (60.9)	
Rectum	21 (39.6)	12 (40.0)	9 (39.1)	
Metastatic site				
Liver	37	20	17	NS
Lung	21	11	10	
Other	22	9	13	
Number of metastatic site				NS
1	30 (56.7)	19 (63.3)	11 (47.8)	
> 1	23 (43.4)	11 (36.7)	12 (52.2)	
Treatment regimen				NS
First line	39 (73.6)	23 (76.7)	16 (69.6)	
Third line	14 (26.4)	7 (23.3)	7 (30.4)	

*NS* not significant

* Fisher exact *p*-value

^a^Well-differentiated/moderately-differentiated

^b^Poorly-differentiated

**Fig. 3:**
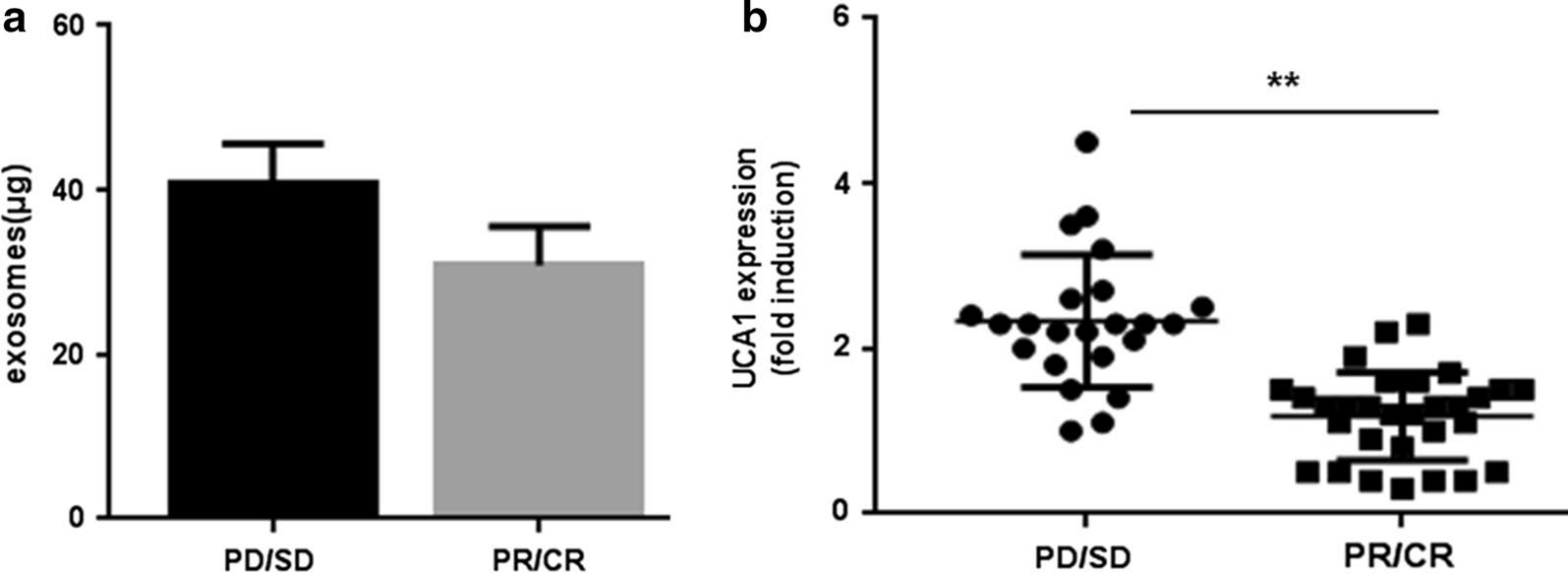
Circulating UCA1-containing exosomes predict the clinical outcome of cetuximab therapy in CRC patients. **a** The quantity of exosomes was higher in PD/SD patients (n = 23) than in PR/CR patients (n = 30), but this difference was not significant. **b** qRT-PCR assays showed a higher UCA1 expression in circulating serum exosomes in PD/SD patients than in those in PR/CR patients (p < 0.01)

As shown in Fig. 3a, in equal amounts of serum, the quantity of exosomes was higher (1.3-fold) in patients who did not respond to cetuximab therapy (PD/SD) than in patients who responded to treatment (PR/CR), but this difference was not significant (p > 0.05). Interestingly, serum exosomal UCA1 expression was significantly higher in the PD/SD group than in the PR/CR group (p < 0.01; Fig. 3b), suggesting that high exosomal UCA1 expression levels might predict increased resistance to cetuximab therapy.

### UCA1 can be transferred from resistant cells to sensitive cells through exosomes

**Fig. 4:**
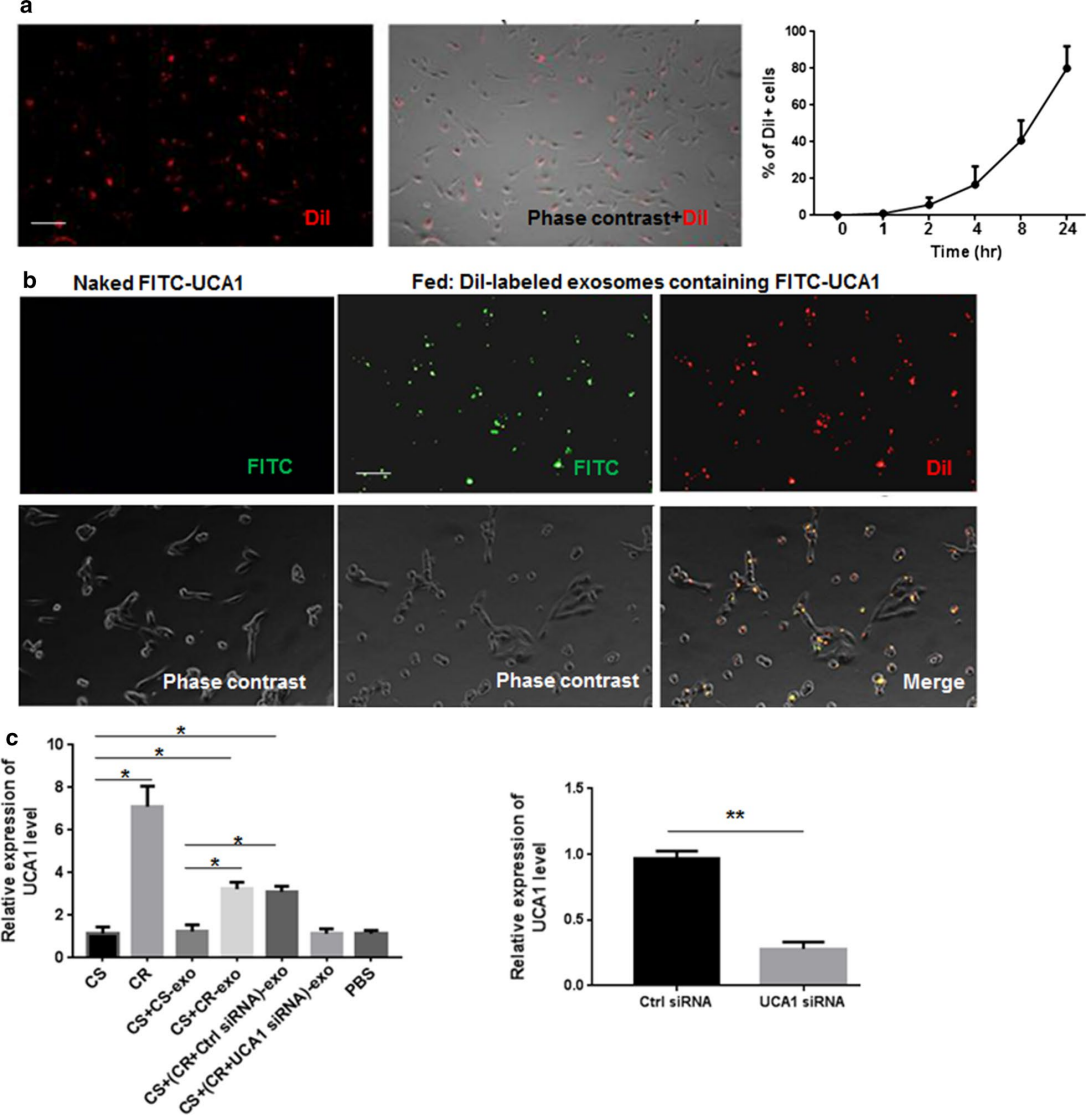
Intercellular transfer of UCA1 by exosomes. **a** Left: fluorescence images of Caco2-CS cells incubated with Dil-labeled (red) exosomes derived from Caco2-CR cells for 48 h. Right: flow cytometry analysis of Dil-positive Caco2-CS cells after incubation with Dil-labeled exosomes for the indicated time. **b** Fluorescence images of Caco2-CS cells 48 h after incubation with Dil-labeled (red) exosomes derived from Caco2-CR cells electroporated with FITC-UCA1 (green) or incubated with naked FITC-UCA1. Representative fluorescence images and phase contrast images are shown. Scale bar, 500 μm. **c** Left: qRT-PCR analysis of UCA1 in Caco2-CS cells (CS) 48 h after incubation with the indicated exosomes. PBS was used as the control group (n = 3). Caco2-CR (CR) cells were used as a positive control. Right: qRT-PCR analysis of UCA1 in UCA1-knockdown and control Caco2-CR cells (n = 3)

Previous studies have suggested that UCA1 is a resistance factor for anticancer drugs [17]. In addition, cell-secreted exosomes and capped nucleic acid cargo can be internalized by neighboring cells [22, 23]. Accordingly, we reasoned that exosomes may carry UCA1 and mediate its intercellular transport. First, CR-exo were labeled with the dye PKH26 and then added to the culture medium of Caco2-CS cells in order to investigate whether exosomes are internalized by recipient cells. Microscopic images showed that labeled exosomes were internalized by Caco2-CS cells in a time-dependent manner after coincubation (Fig. 4a). To further confirm that UCA1 could be transferred to recipient cells via exosomes, Caco2-CS cells were electroporated with fluorescein isothiocyanate (FITC)-UCA1, then exosomes were isolated and labeled with Dil. As shown in Fig. 4b, we observed that colocalization of FITC and Dil was in most recipient cells, whereas no internalization of naked FITC-UCA1 was found in recipient cells after incubation with labeled exosomes. Moreover, to evaluate whether the transport of exosomes derived from drug-resistant cells altered UCA1 levels, we coincubated Caco2-CS cells with CS-exo or CR-exo for 48 h, and UCA1 levels in the Caco2-CS, Caco2-CS + CS-exo, Caco2-CS + CR-exo and Caco2-CR groups were analyzed by qRT-PCR. The level of UCA1 increased in the Caco2-CS + CR-exo group compared with the Caco2-CS and Caco2-CS + CS-exo groups but did not increase in UCA1-knockdown-resistant cells; however, UCA1 levels did not significantly change in the Caco2-CS + CS-exo group compared with the Caco2-CS group (Fig. 4c). These results show that exosomes carrying UCA1 content from cetuximab-resistant cells can be transferred to recipient sensitive cells.

### Exosomes derived from cetuximab-resistant cells can transmit drug resistance to sensitive cells and alter UCA1 expression

**Fig. 5:**
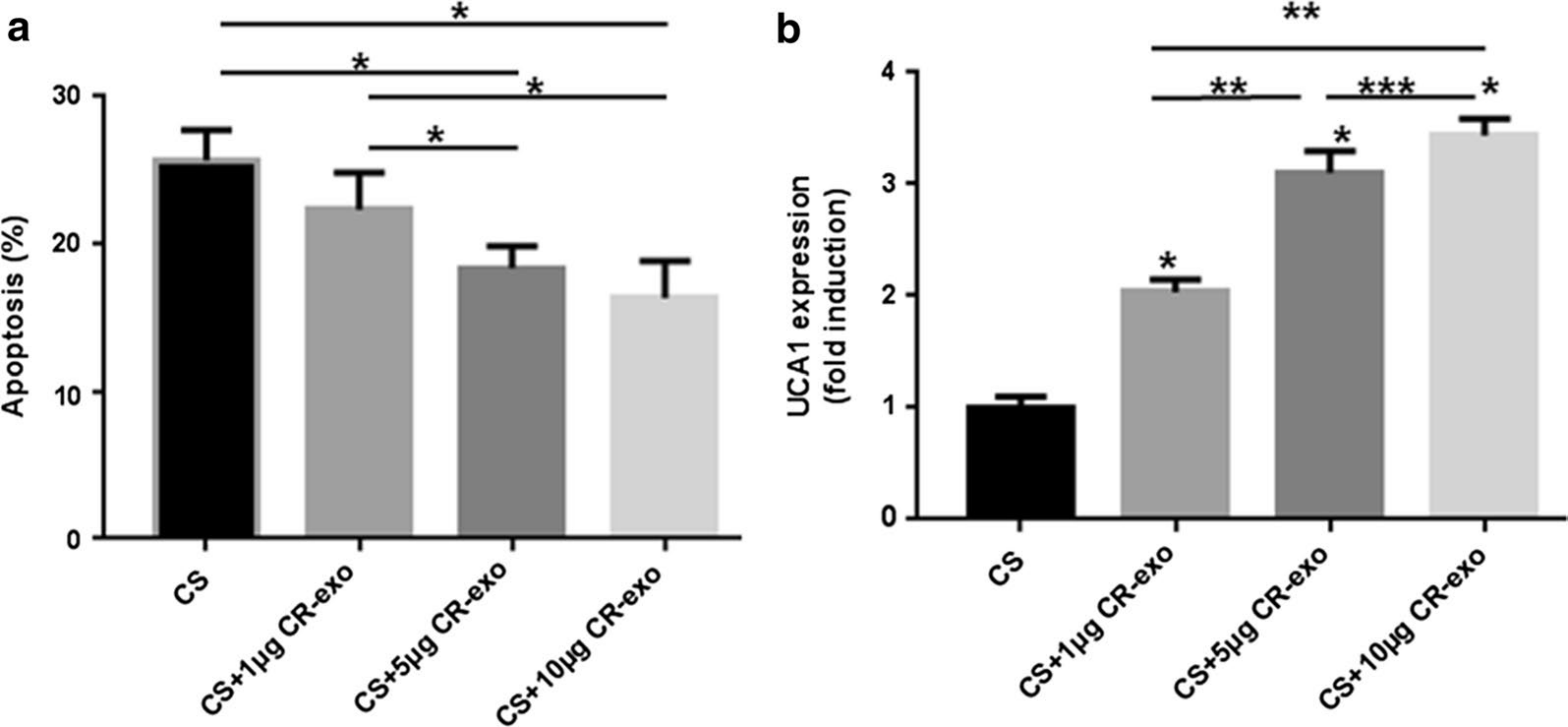
Exosomes derived from cetuximab-resistant cells conferred drug resistance to sensitive cells and altered UCA1 expression. Caco2-CS cells were treated with cetuximab for 48 h before flow cytometry analysis after coculture with or without CR-exo for 24 h. The apoptotic rates of Caco2-CS cells exposed to cetuximab were analyzed in four samples: Caco2-CS cells (CS), Caco2-CS cells coincubated with 1 μg CR-exo (CS + 1 μg CR-exo), Caco2-CS cells coincubated with 5 μg CR-exo (CS + 5 μg CR-exo), and Caco2-CS cells coincubated with 10 μg CR-exo (CS + 10 μg CR-exo). **a** The results are representative of three independent experiments (the mean apoptotic rate ± SD). Apoptotic rates in the CS + 10 μg CR-exo and CS + 5 μg CR-exo groups were significantly increased compared with those in the CS and CS + 1 μg CR-exo groups (*p < 0.05), while the difference between the CS + 5 μg CR-exo and CS + 10 μg CR-exo groups was not significant. **b** The UCA1 expression level significantly increased in a dose-dependent manner in the above four groups of CR-exo-treated cells: groups incubated with greater amounts of CR-exo showed higher UCA1 expression. Different symbols indicated the significant differences (p < 0.05) (*compared with CS; **compared with CS + 1 μg CR-exo; ***compared with CS + 5 μg CR-exo)

In the end, we investigated the role of UCA1-containing exosomes in the transfer of cetuximab resistance. We added different amounts of CR-exo to the medium of Caco2-CS cells (CS + 1/5/10 μg CR-exo, Caco2-CS cells were used as the control group). After 24 h of culture, we added serial dilutions of cetuximab to all groups. Two days later, we found a reduction in apoptosis in the groups coincubated with CR-exo (CS + 1/5/10 μg CR-exo) compared with the CS group (p < 0.05), indicating the increased resistance to cetuximab (Fig. 5a). Interestingly, the apoptosis rates of Caco2-CS cells incubated with CR-exo were inversely dependent on the CR-exo dose. Moreover, UCA1 levels significantly increased in CR-exo-supplemented cells in a dose-dependent manner (Fig. 5b). Thus, we reasoned that UCA1 might play a role in cetuximab resistance and that exosomal UCA1 might transmit cetuximab resistance in CRC cells, resulting in greater cetuximab resistance in recipient cells.

## Discussion

Cetuximab is one of the most widely used EGFR inhibitors in the treatment of mCRC patients with wild-type KRAS status. However, primary or acquired resistance to cetuximab often occurs during targeted therapy. To optimize individualized cetuximab therapy in CRC patients, it is essential to identify possible indicators of the response to cetuximab therapy.

The lncRNA UCA1 has been found to reactive and is a promising diagnostic and prognostic biomarker in various malignant tumors [15]. Furthermore, many studies have shown that UCA1 induces drug resistance in various cancers [17]. The role of UCA1 in CRC drug resistance has also been described [24]. Bian et al. [24] showed that ectopic expression of UCA1 increased the chemoresistance of CRC cells to 5-fluorouracil (5-FU), whereas knockdown of UCA1 enhanced apoptosis in HCT116 cells. Mechanistically, UCA1 acts as a sponge for miR-204-5p and upregulates the level of several target genes of miR-204-5p, suggesting the important role of UCA1 in chemoresistance. Therapeutic monitoring using human blood is considered a noninvasive and convenient method, and circulating lncRNAs as biomarkers for predicting therapeutic efficacy in cancer has been investigated for years [19, 25]. However, the potential of using UCA1 levels in body fluid samples as a predictive biomarker of drug resistance remains to be confirmed.

Increasing evidence indicates that the tumor microenvironment can also contributes to changes in tumor characteristics [26, 27], such as resistance to some anticancer drugs [28, 29]. Exosomes are a promising tumor-derived material for the characterization of some tumor behaviors [30, 31]. Sugimachi et al. [32] showed that serum exosomal miRNAs could be potential novel biomarkers for predicting tumor recurrence and prognosis. Furthermore, Ma et al. [33] demonstrated that TrpC5-containing extracellular vesicles might be a potential biomarker for chemoresistant breast cancer. Here, we sought to investigate whether exosomal UCA1 is involved in the drug resistance process and to determine the significance of the transfer of cetuximab-resistance properties in CRC. In the current study, we first clarified the upregulation of UCA1 levels in cetuximab-resistant cells and their exosomes. Interestingly, it was observed that UCA1 detectable in cells and serum is concentrated in exosomes. In fact, the increase in exosomes is more evident than in cells, and therefore, purifying exosomes could improve the sensitivity of circulating UCA1 detection. Therefore, we reason that exosome-mediated transfer of UCA1 might be an important mechanism of acquitted cetuximab resistance in CRC cells. We also systematically evaluated the stability of exosomal UCA1, and the results indicated that exosomal UCA1 is evidently stable when subjected to severe conditions, suggesting that the exosomal UCA1 can be protected by exosome membrane. Furthermore, we observed that circulating UCA1-containing exosomes could predict the clinical outcome of cetuximab therapy in CRC patients. Although the quantity of exosomes was nearly equal in the PD/SD and PR/CR groups, interestingly, UCA1 level was markedly higher in PD/SD patients than in PR/CR patients. As exosomes are important players in intercellular communication processes [34–36], we further investigated whether exosomes are involved in drug resistance process and evaluated the significance of the transfer of drug-resistance properties in CRC. We observed that exosomes derived from cetuximab-resistant cells can transmit drug resistance to sensitive cells and cause changes in UCA1 level. These findings further support for the hypothesis that analyzing exosomal lncRNA expression in blood as a biomarker become ever more feasible and that UCA1-containing exosomes can predict cetuximab therapy efficacy in CRC patients.

The molecular mechanisms by which most lncRNAs function remain to be elucidated. LncRNAs play roles in signaling and can act as decoys, guides or scaffolds by specifically binding to target DNAs, RNAs and proteins. While these mechanisms are not mutually exclusive, some lncRNAs may have one or more roles. In different cancer cells, UCA1 has been demonstrated to bind to several miRNAs [24, 37–43]. In addition to its oncogenic effect, UCA1 also exerts a regulatory effect on drug resistance in several types of cancer. For example, UCA1 can induce acquired resistance to EGFR-tyrosine kinase inhibitors (TKIs) in EGFR-mutant nonsmall cell lung cancer by activating the AKT/mTOR pathway [44]. It can also increase the chemoresistance of bladder cancer cells by activating the Wnt signaling pathway in a Wnt6-dependent manner [45]. Knockdown of UCA1 in adriamycin-resistant SGC7901/ADR cells can significantly decrease resistance [46]. The major limitation of this study is that downstream regulation of UCA1 in cetuximab-resistant cells was not investigated. We hypothesized that some molecular effectors, such as miRNA, might be important targets of UCA1, leading to dysregulation of the signaling pathway in cetuximab resistance. However, further studies are required to validate our hypothesis.

## Conclusions

In summary, this is the first demonstration that exosomal UCA1 has the key characteristics of a tumor marker: it can be assayed in a noninvasive manner, and it is relatively abundant and stable. Moreover, we explored exosomal UCA1 as a biomarker for predicting drug sensitivity in patients who were treated with cetuximab. UCA1-containing exosomes appear to be essential for conferring cetuximab resistance. Although some factors involving in drug resistance has been widely studied, our findings on UCA1 open important possibilities for future clinical application. Thus, both basic and clinical researchers should engage in multidisciplinary efforts monitoring of cetuximab resistance. As mentioned above, UCA1 is a potential good broad-spectrum biomarker for cancer diagnosis, prognosis or prediction of therapeutic response. However, no UCA1 products have been identified in clinical trials. Further preclinical and clinical studies will highlight and better elucidate the role of UCA1 in anticancer drug resistance and may lead to future investigations of the various clinical applications of UCA1.

## Abbreviations

CRC: colorectal cancer
EGFR: epidermal growth factor receptor
MAb: monoclonal antibody
mCRC: metastatic CRC
lncRNAs: long noncoding RNAs
UCA1: urothelial carcinoma associated 1
PR: partial response
CR: complete response
PD: progressive disease
SD: stable disease
DMEM: Dulbecco’s Modified Eagle’s Medium
CCK8: Cell Counting Kit 8
PBS: phosphate-buffered saline
TEM: transmission electron microscopy
qRT-PCR: quantitative real-time reverse transcription-polymerase chain reaction
SDS: sodium dodecyl sulfate
ECL: enhanced chemiluminescence
SiRNA: small interfering RNA
PI: propidium iodide
SD: standard deviation
FITC: fluorescein isothiocyanate
